# On the Composability of Statistically Secure Random Oblivious Transfer

**DOI:** 10.3390/e22010107

**Published:** 2020-01-16

**Authors:** Rafael Dowsley, Jörn Müller-Quade, Anderson C. A. Nascimento

**Affiliations:** 1 Department of Computer Science, Bar-Ilan University, Ramat Gan 5290002, Israel; 2 Institute of Theoretical Informatics, Karlsruhe Institute of Technology, 76131 Karlsruhe, Germany; 3 School of Engineering & Technology, University of Washington Tacoma, Tacoma, WA 98402, USA

**Keywords:** random oblivious transfer, unconditional security, universal composability

## Abstract

We show that random oblivious transfer protocols that are statistically secure according to a definition based on a list of information-theoretical properties are also statistically universally composable. That is, they are simulatable secure with an unlimited adversary, an unlimited simulator, and an unlimited environment machine. Our result implies that several previous oblivious transfer protocols in the literature that were proven secure under weaker, non-composable definitions of security can actually be used in arbitrary statistically secure applications without lowering the security.

## 1. Introduction

Oblivious transfer (OT) [1] is a primitive of central importance in modern cryptography and implies secure computation [2,3]. Several flavors of OT were proposed, but they are all equivalent [4]. In this work, we focus on the so-called one-out-of-two random oblivious transfer. This is a two-party primitive in which a sender (Alice) gets two uniformly random bits $b_{0}$, $b_{1}$ and a receiver (Bob) gets a uniformly random choice bit *c* and $b_{c}$. Bob remains ignorant about $b_{\bar{c}}$. On the other hand, Alice cannot learn the choice bit *c*.

Many OT protocols are known, based on various assumptions (both computational and physical) and achieving diverse notions of security. However, weaker security notions do not guarantee security when multiple copies of the protocol are executed, or when the OT protocols are used as building blocks within other protocols. This is an unsatisfactory state of affairs, as the major utility of OT is in the modular designing of larger protocols. Following the simulation paradigm used in [5] to define the seminal notion of zero-knowledge proofs of knowledge, many simulation-based definitions of security for multi-party protocols were proposed (e.g., [2,6]) and they guarantee that the protocols are sequentially composable [7]; however, this paradigm of security does not guarantee general composability of the protocols. UC-security [8] emerges as a very desirable notion of security for OT since it guarantees that the security of the protocol holds even when the OT scheme is concurrently composed with an arbitrary set of protocols. UC-security is a very powerful notion of security that allows to fully enjoy the nice properties of OT within other protocols.

Some questions about the equivalence of list-based and composable security notions in the case of statistically secure protocols were studied [9,10]. In general, these security notions are not equivalent [10]. Therefore, it is an interesting question to study if there are restricted scenarios where this equivalence holds.

*Our Results:* In this paper, we show that random OT protocols that are based on certain stateless two-party functionalities and that match a certain list of information-theoretical security properties (i.e., statistical equalities involving the statistical information) are not only secure in a simulation-based way, but are actually UC-secure with an unlimited adversary, an unlimited simulator, and an unlimited environment machine. Note that Random OT can be straightforwardly used to obtain OT for arbitrary inputs in a secure and composable way [11,12]. Note also that most OT protocols based on two-party stateless functionalities already internally run a random OT protocol and then use derandomization techniques to obtain OT for arbitrary inputs. We think that this approach is interesting because, in this scenario, a protocol designer can worry only about meeting the list-based security notion and the protocol inherits the UC-security. The setting studied in this paper covers the case of statistically secure protocols based on noisy channels, cryptogates, and pre-distributed correlated data. As a consequence of our result, several previously proposed protocols implementing oblivious transfer that were proven secure in weaker models automatically have their security upgraded to a simulation-based, composable one for free [11,13,14,15,16,17,18,19,20,21,22,23,24,25,26].

### Related Work

OT can be constructed based both on generic computational assumptions such as the existence of enhanced trapdoor permutations [27,28] and on the computational hardness of many specific problems such as factoring [1], Diffie–Hellman [29,30], Learning with Errors (LWE) [31], variants of Learning Parity with Noise (LPN) [32], and McEliece assumptions [33,34]. However, the focus of this work is on statistically secure OT. When aiming for statistical security, OT can be based on noisy channels [13,14,15,16,17,18,19,20,21], cryptogates [22,23], pre-distributed correlated data [11,18,24], the bounded storage model [35,36,37,38], and on hardware tokens [25,26].

Canetti and Fischlin [39] showed that OT cannot be UC-realized in the plain model, thus additional setup assumptions are required. UC-secure OT protocols were initially constructed in the common reference string (CRS) model [31,40,41]. In the CRS model there exists an honestly generated random string that is available to the parties (the simulator can generate its own string as long as it looks indistinguishable from the honestly generated one). In the public key infrastructure model, Damgård and Nielsen [42] proposed an OT protocol that is UC-secure against adaptive adversaries under the assumption that threshold homomorphic encryption exists. Katz [43] proved that two-party and multi-party computation are possible assuming a tamper-proof hardware.

The question about the equivalence of list-based and composable security definitions for statistically secure protocols was previously addressed in [9,10], where it was proven that the equivalence does not hold in general. In [44], it was proven that perfectly secure OT protocols according to a list of properties are *sequentially* composable, this result being extended to statistical security in [45].

It was shown that, for statistically secure commitment schemes based on two-party stateless primitives, a list-based security definition actually implies UC-security [46]. While this result implies the possibility of building UC-secure OT protocols based on these commitment protocols, this is not the most efficient way of obtaining OT and it does not prove any additional security property about the existing OT protocols.

Even if the resources available to the parties to implement OT are asymmetric, Wolf and Wullschleger [12] showed a very simple way to reverse the OT’s direction (indeed, all complete two-party functionalities are reversible, as proved recently by Khurana et al. [47]).

## 2. Preliminaries

### 2.1. Notation

Domains of random variables are denoted by calligraphic letters, the random variables by uppercase letters, and the realizations by lowercase letters. For random variables *X* over X and *Y* over Y, $P_{X}:X\to \left[0,1\right]$ with $\sum_{x\in X}P_{X}\left(x\right)=1$ denotes the probability distribution of *X*, $P_{X}\left(x\right):=\sum_{y\in Y}P_{XY}\left(x,y\right)$ the marginal probability distribution, and $P_{X|Y}\left(x|y\right):=P_{XY}\left(x,y\right)/P_{Y}\left(y\right)$ the conditional probability distribution if $P_{Y}\left(y\right)\neq 0$. The statistical distance $\delta (P_{X},P_{Y})$ between $P_{X}$ and $P_{Y}$ with alphabet X is given by

$$\delta \left(P_{X},P_{Y}\right)=\max_{S\subseteq X}\left|\sum_{x\in S}P_{X}\left(x\right)-P_{Y}\left(x\right)\right|.$$

We say $P_{X}$ and $P_{Y}$ are ε-close if $\delta (P_{X},P_{Y})\leq \varepsilon$. Following Crépeau and Wullschleger [45], let the statistical information of *X* and *Y* given *Z* be defined as

$$I_{S}\left(X;Y|Z\right)=\delta \left(P_{XYZ},P_{Z}P_{X|Z}P_{Y|Z}\right).$$

### 2.2. The UC Framework

Here, we briefly review the main concepts of the UC framework, for more details please refer to the original work of Canetti [8]. In the UC framework, the security of a protocol to carry out a certain task is ensured in three phases:

1. One formalizes the framework, i.e., the process of executing a protocol in the presence of an adversary and an environment machine.
2. One formalizes an ideal protocol for carrying out the task in an ideal protocol using a “trusted party”. In the ideal protocol, the trusted party captures the requirements of the desired task and the parties do not communicate among themselves.
3. One proves that the real protocol emulates the ideal protocol, i.e., for every adversary in the real model, there exists an ideal adversary (also known as the simulator) in the ideal model such that no environment machine can distinguish if it is interacting with the real or the ideal world.

The environment in the UC framework represents all activity external to the running protocol, thus it provides inputs to the parties running the protocol and receives the outputs that the parties generate during the execution of the protocol. As stated above, the environment also tries to distinguish between attacks on real executions of the protocol and simulated attacks against the ideal functionality. If no environment can distinguish the two situations, the real protocol emulates the ideal functionality. Proving that a protocol is secure in the UC framework provides the following benefits:

1. The ideal functionality describes intuitively the desired properties of the protocol.
2. The protocols are secure under composition.
3. The security is retained when the protocol is used as a sub-protocol to replace an ideal functionality that it emulates.

#### 2.2.1. The Ideal World

An ideal functionality F represents the desired properties of a given task. Conceptually, F is treated as a local subroutine by the several parties that use it, and thus the communication between the parties and F is supposedly secure (i.e., messages are sent by input and output tapes). The ideal protocol also involves a simulator S, an environment Z on input *z*, and a set of dummy parties that interacts as defined below. Whenever a dummy party is activated with input *x*, it writes *x* onto the input tape of F. Whenever the dummy party is activated with value *x* on its subroutine output tape, it writes *x* on subroutine output tape of Z. The simulator S has no access to the contents of messages sent between dummy parties and F, and it should send corruption messages directly to F, which is responsible for determining the effects of corrupting any dummy party. The ideal functionality receives messages from the dummy parties by reading its input tape and sends messages to them by writing to their subroutine output tape. In the ideal protocol, there is no communication among the parties. The environment Z can set the inputs to the parties and read their outputs, but cannot see the communication with the ideal functionality.

#### 2.2.2. The Real World

In the real world, the protocol π is executed by parties $P_{1},\ldots ,P_{n}$ with some adversary A and an environment machine Z with input *z*. Z can set the inputs for the parties and see their outputs, but not the communication among the parties. The parties can invoke subroutines, pass inputs to them, and receive outputs from them. They can also write messages on the incoming communication tape of the adversary. These messages may specify the identity of the final destination of the message. A can send messages to any party (A delivers the message). In addition, they may use the ideal functionalities that are provided to the real protocol. A can communicate with Z and the ideal functionalities that are provided to the real protocol. A also controls the corrupt parties (the environment always knows which parties are corrupted).

#### 2.2.3. The Adversarial Model

The network is asynchronous without guaranteed delivery of messages. The communication is public, but authenticated (i.e., the adversary cannot modify the messages). The adversary controls the corrupted parties. Any number of parties can be corrupted, but we consider static corruptions, i.e., the corruptions happen before the beginning of the protocol. Finally, the adversary, the environment, and the simulator are allowed unbounded complexity. This assumption on the computational power of the simulator somehow weakens our result as the composition theorem cannot be applied several times if the real adversary were restricted to polynomial time, because the “is at least as secure as” relation can no longer be proven to be transitive. However, arbitrary composition is allowed when considering statistically secure protocols and this situation is common in the literature when proving general results on the composability of statistically secure protocols [9,10,44,45].

#### 2.2.4. Realizing an Ideal Functionality

A protocol π statistically UC-realizes an ideal functionality F if for any real-life adversary A there exists a simulator S such that no environment Z, on any input *z*, can tell with non-negligible probability whether it is interacting with A and parties running π in the real-life process, or it is interacting with S and F in the ideal protocol. This means that, from the point of view of the environment, running protocol π is statistically indistinguishable from the ideal world with F.

#### 2.2.5. The Oblivious Transfer Functionality

**Figure 1:**
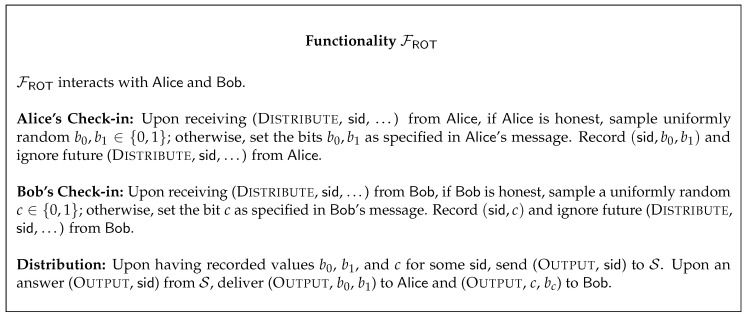
The one-out-of-two bit random oblivious transfer functionality.

We present in Figure 1 the one-out-of-two bit random oblivious transfer functionality $F_{\mathrm{ROT}}$. The sender is denoted by Alice and the receiver by Bob.

### 2.3. Setup Assumption

**Figure 2:**
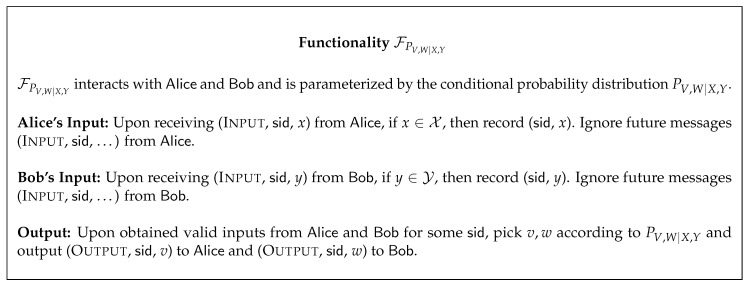
The functionality, given valid inputs, samples outputs according to the conditional probability distribution and delivers the outputs to Alice and Bob.

In this work, we consider the scenario in which Alice and Bob have access to the functionality $F_{P_{V,W|X,Y}}$ that given inputs x∈X from Alice and y∈Y from Bob samples the outputs v∈V and w∈W according to the conditional probability distribution $P_{V,W|X,Y}$, and gives the outputs *v* and *w* to Alice and Bob, respectively. The functionality $F_{P_{V,W|X,Y}}$ is described in Figure 2. We remark that this functionality captures several cryptographic primitives that have been previously used for obtaining secure oblivious transfer, including noisy channels and pre-distributed correlated data. Similar cryptographic primitives have appeared in the literature under a different terminology [48,49].

## 3. Random Oblivious Transfer Based on Statistically Secure Two Party Stateless Functionalities

In this section, we define a list-based security model for random OT protocols that achieve statistical security by using $F_{P_{V,W|X,Y}}$ as a setup assumption. Alice and Bob have two resources available between them:

- a bidirectional authenticated noiseless channel denoted as $F_{\mathrm{AUTH}}$, and
- the functionality $F_{P_{V,W|X,Y}}$.

We stress we are not describing a specific protocol in this section. Rather, we are providing a general framework and security definitions that encompasses any protocol that implements OT based on two-party stateless functionalities and has statistical security. In the following, we model the probabilistic choices of Alice by a random variable ${\mathrm{coins}}_{\mathrm{Alice}}$ and those of Bob by a random variable ${\mathrm{coins}}_{\mathrm{Bob}}$, so that we can use deterministic functions in the protocol. As usual, we assume that the noiseless messages exchanged by the players and their personal randomness are taken from ${\left\{0,1\right\}}^{*}$. We now describe a generic protocol π that is general enough to captures any protocol implementing OT based on two-party stateless functionalities with statistical security.

Protocol π

Alice and Bob interact and in the end of the execution Alice gets $\left(b_{0},b_{1}\right)$ and Bob gets $\left(c,b_{c}\right)$, for $b_{0},b_{1},c\in \left\{0,1\right\}$ picked uniformly at random. The security parameter is *n*, and determines how many times the parties can use the functionality $F_{P_{V,W|X,Y}}$: in the *i*th round, Alice and Bob input symbols $x_{i}$ and $y_{i}$ to the functionality $F_{P_{V,W|X,Y}}$, which generates the outputs $v_{i}$ and $w_{i}$ according to $P_{V,W|X,Y}$ and delivers them to Alice and Bob, respectively. Let $x^{i}$, $y^{i}$, $v^{i}$, and $w^{i}$ denote the vectors of these variables until *i*th round. The parties can use $F_{\mathrm{AUTH}}$ at any moment. Let trans denote all the noiseless messages exchanged between the players.

We call the view of Bob all the data in his possession, i.e., $y^{n},w^{n},c,{\mathrm{coins}}_{\mathrm{Bob}}$, and trans, and denote it by ${\mathrm{view}}_{\mathrm{Bob}}$. ${\mathrm{view}}_{\mathrm{Alice}}$ is defined similarly. We denote the output of the (possibly malicious) parties Alice and Bob by ${\mathrm{output}}_{\mathrm{Alice}}$ and ${\mathrm{output}}_{\mathrm{Bob}}$, respectively. The list-based definition of security that is henceforth considered in this paper follows the lines of Crépeau and Wullschleger [45]. The protocol is said to be secure if there exists an ϵ that is a negligible function of the security parameter *n* and is such that the following properties are satisfied:

Correctness 

If both parties are honest, then ${\mathrm{output}}_{\mathrm{Alice}}=\left(b_{0},b_{1}\right)$ and ${\mathrm{output}}_{\mathrm{Bob}}=\left(c,d\right)$ for d∈{0,1} and uniformly random $b_{0},b_{1},c\in \left\{0,1\right\}$. Additionally,

$$\mathrm{Pr}[D=B_{C}]\geq 1-\epsilon .$$

Security for Alice

If Alice is honest, then ${\mathrm{output}}_{\mathrm{Alice}}=\left(b_{0},b_{1}\right)$ for uniformly random $b_{0},b_{1}\in \left\{0,1\right\}$ and there exists a random variable *C* such that

$$I_{S}\left(B_{0},B_{1};C\right)\leq \epsilon$$

and

$$I_{S}\left(B_{0},B_{1};{\mathrm{output}}_{\mathrm{Bob}}|C,B_{C}\right)\leq \epsilon .$$

Security for Bob

If Bob is honest, then ${\mathrm{output}}_{\mathrm{Bob}}=\left(c,d\right)$ for d∈{0,1} and uniformly random c∈{0,1}; and

$$I_{S}\left(C;{\mathrm{output}}_{\mathrm{Alice}}\right)\leq \epsilon .$$

## 4. UC-Security Implication

In this section, we address the question of whether random OT protocols that are secure according to the definitions of Section 3 also enjoy statistical UC-security. We show that this is indeed the case. Intuitively, this follows from the fact that the security in those protocols is based on the correlated randomness that is provided by the functionality $F_{P_{V,W|X,Y}}$ to Alice and Bob. Since in the ideal world the simulator controls $F_{P_{V,W|X,Y}}$, it can leverage this knowledge in order to extract the outputs of the corrupted parties and forward them to the random oblivious transfer functionality $F_{\mathrm{ROT}}$, thus allowing the ideal execution to be indistinguishable from the real execution from the environment’s point of view. First, we prove some lemmas that are used below to prove the main result of this work.

We first show that, in any random OT protocol that is secure according to the definitions of Section 3, if Bob is honest, then given Alice’s input to and output from the functionality $F_{P_{V,W|X,Y}}$ and all the noiseless communication exchanged by Alice and Bob through $F_{\mathrm{AUTH}}$, it is possible to extract both outputs that Bob would get with c=0 and c=1 in the random OT protocol.

**Lemma** **1.** *Let π be a random OT protocol that is secure according to the definitions of Section 3 and let Bob be honest. Given Alice’s input to and output from $F_{P_{V,W|X,Y}}$ and all the noiseless communication exchanged by Alice and Bob through $F_{\mathrm{AUTH}}$ during the execution of π, with overwhelming probability, it is possible to extract the output that Bob would get both in the case that c=0 and c=1.*

**Proof.**  Let us consider an execution of the protocol π in which Bob has random coins ${\mathrm{coins}}_{\mathrm{Bob}}$ and gets ${\mathrm{output}}_{\mathrm{Bob}}=\left(c,d\right)$ for d∈{0,1} and uniformly random c∈{0,1} (as the protocol is secure according to the definitions of Section 3). Denote by *m* the set of messages exchanged between Alice and $F_{P_{V,W|X,Y}}$ concatenated with the noiseless messages between Bob and Alice. We claim that there should exist $\bar{{\mathrm{coins}}_{\mathrm{Bob}}}\neq {\mathrm{coins}}_{\mathrm{Bob}}$ so that, for the same *m*, if Bob executed the protocol with $\bar{{\mathrm{coins}}_{\mathrm{Bob}}}$ he should have been able with overwhelming probability to get $\bar{{\mathrm{output}}_{\mathrm{Bob}}}=\left(\bar{c},\bar{d}\right)$ with $\bar{c}\neq c$ and $\bar{d}\in \left\{0,1\right\}$. If that were not the case, Alice would know that Bob is unable to obtain a valid output $\bar{d}$ when the choice bit is $\bar{c}$, thus gaining knowledge on the choice bit and breaking the protocol security. Given that

$$I_{S}\left({\mathrm{output}}_{\mathrm{Alice}};C\right)\leq \epsilon ,$$

we get

$$\delta (P_{{\mathrm{output}}_{\mathrm{Alice}}C},P_{{\mathrm{output}}_{\mathrm{Alice}}}P_{C})\leq \epsilon ,$$

and so there are events $E_{1}$ and $E_{2}$ such that

$$\mathrm{Pr}\left[E_{1}\right]=\mathrm{Pr}\left[E_{2}\right]=1-\epsilon \;\mathrm{and}$$

$$P_{{\mathrm{output}}_{\mathrm{Alice}}C|E_{1}}=P_{{\mathrm{output}}_{\mathrm{Alice}}|E_{2}}P_{C|E_{2}}.$$

Therefore, if $E_{1}$ and $E_{2}$ happen, then ${\mathrm{output}}_{\mathrm{Alice}}$ does not provide information about *C* and $\bar{{\mathrm{coins}}_{\mathrm{Bob}}}$ should exist. Thus, given *m* we are left with an extraction procedure. One just computes ${\mathrm{coins}}_{\mathrm{Bob}}$ and $\bar{{\mathrm{coins}}_{\mathrm{Bob}}}$ that for this *m* produce outputs ${\mathrm{output}}_{\mathrm{Bob}}$ and $\bar{{\mathrm{output}}_{\mathrm{Bob}}}$, respectively, and simulates the protocol execution for each specified Bob’s randomness. □

We now prove that, given access to the messages that Bob exchanges with Alice and $F_{P_{V,W|X,Y}}$, there is a point in the protocol execution in which it is possible to extract the choice bit *c* and still equivocate $b_{0},b_{1}$ to any value, i.e., it is possible to find an Alice’s view that is compatible with the current view of Bob and the new values of $b_{0}$ and $b_{1}$.

**Lemma** **2.** *Let π be a random OT protocol that is secure according to the definitions of Section 3 and let Alice be honest. Given access to all messages that Bob’s exchanges with $F_{P_{V,W|X,Y}}$ and all the noiseless communication exchanged by Alice and Bob through $F_{\mathrm{AUTH}}$ during the execution of π, with overwhelming probability it is possible to extract the choice bit c at some point of the execution of the protocol π. Additionally, at this point, it is still possible to change $b_{0}$ and $b_{1}$ to any desired values.*

**Proof.**  We first prove that there is a point in the protocol execution where we can extract the choice bit given the messages that Bob exchanged with Alice and the functionality $F_{P_{V,W|X,Y}}$. Let *m* denote these messages in a given protocol execution. Let M(0) denote the set of messages that allow Bob to obtain the bit $b_{0}$ with overwhelming probability (the probability taken over ${\mathrm{coins}}_{\mathrm{Alice}}$, ${\mathrm{coins}}_{\mathrm{Bob}}$ and the randomness of $F_{P_{V,W|X,Y}}$). Let M(1) be defined similarly for $b_{1}$. From the security for Alice, we have that

$$I_{S}\left(B_{0},B_{1};C\right)\leq \epsilon$$

and

$$I_{S}\left(B_{0},B_{1};{\mathrm{output}}_{\mathrm{Bob}}|C,B_{C}\right)\leq \epsilon ,$$

and so we get that *m* with overwhelming probability (over ${\mathrm{coins}}_{\mathrm{Alice}}$ and the randomness of $F_{P_{V,W|X,Y}}$) cannot be in both M(0) and M(1), since this fact would imply that the resulting protocol would be insecure for Alice. This fact gives us a procedure for obtaining the choice bit *c* given *m*. We just check if *m* is in M(0) or M(1).

We now turn to the equivocation property. From the previous reasoning, we know that there should exist a point in the protocol where Bob sends a message to Alice that fixes the choice bit (i.e., the choice bit can be extracted from his messages from/to Alice and $F_{P_{V,W|X,Y}}$). Let *i* be the index of such message. Suppose the *i*th message is the very last one in the protocol. Then, Bob has all the information necessary to compute his output even before sending the *i*th message. As the choice bit is only fixed in the next message, Bob should be able to compute both $b_{0}$ and $b_{1}$, breaking Alice’s security. Thus, the *i*th message should not be the last one. The same reasoning implies that, from Bob’s point of view, none of Alice’s outputs $b_{0}$ and $b_{1}$ can be fixed before the *i*th message: (1) if both $b_{0}$ and $b_{1}$ are fixed from Bob’s point of view before the *i*th message, then he could obtain both $b_{0}$ and $b_{1}$ and break the security according to the definitions of Section 3; and (2) if only $b_{i}$ is fixed, then Bob can still change his choice to c=1−i and obtain both $b_{0}$ and $b_{1}$, thus breaking the security definition of Section 3. Therefore, we should have that, when the *i*th message is sent by Bob, Alice’s outputs $b_{0}$ and $b_{1}$ are still equivocable. □

We now use two lemmas to prove our main result:

**Theorem** **1.** *Any random OT protocol based on $F_{\mathrm{AUTH}}$ and $F_{P_{V,W|X,Y}}$ that is secure according to the definitions of Section 3 also UC-realizes $F_{\mathrm{ROT}}$.*

**Proof.**  We construct the simulator S as follows. S runs a simulated copy of A in a black-box way, plays the role of the ideal functionality $F_{P_{V,W|X,Y}}$, and simulates a copy of the hybrid interaction of π for the simulated adversary A. In addition, S forwards the messages between Z and A. Below, we describe the procedures of the simulator in each occasion:

**Only Alice is corrupted:**S samples the randomness ${\mathrm{coins}}_{\mathrm{Bob}}$ of the simulated Bob and proceeds with the simulated execution of the protocol π by producing his noiseless messages as well as his inputs $y_{i}\in Y$ to $F_{P_{V,W|X,Y}}$. Additionally, once the inputs $x_{i}\in X$ and $y_{i}\in Y$ to $F_{P_{V,W|X,Y}}$ are fixed, S simulates the outputs of the functionality $F_{P_{V,W|X,Y}}$ and sends $v_{i}$ to A. As S plays the role of $F_{P_{V,W|X,Y}}$, when the execution is done, S extracts the output bits $b_{0},b_{1}$ of the corrupted Alice using the result of lemma 1 and forwards $b_{0},b_{1}$ to $F_{\mathrm{ROT}}$. S then allows $F_{\mathrm{ROT}}$ to deliver the output.

**Only Bob is corrupted:**S samples the randomness ${\mathrm{coins}}_{\mathrm{Alice}}$ of the simulated Alice and proceeds with the simulated execution of the protocol π by producing her noiseless messages as well as her inputs $x_{i}\in X$ to $F_{P_{V,W|X,Y}}$. Additionally, once the inputs $x_{i}\in X$ and $y_{i}\in Y$ to $F_{P_{V,W|X,Y}}$ are fixed, S simulates the outputs of the functionality $F_{P_{V,W|X,Y}}$, and sends $w_{i}$ to A. Then, using the result of lemma 2, S extracts the choice bit *c* of the corrupted Bob, inputs *c* to $F_{\mathrm{ROT}}$, receives $b_{c}$, and finishes the simulated protocol execution in such way that the received bit in the hybrid interaction $b_{c}^{\prime }$ is equal to the received bit in the ideal protocol $b_{c}$ with overwhelming probability.

**Neither party is corrupted:**S samples the randomness ${\mathrm{coins}}_{\mathrm{Alice}}$ and ${\mathrm{coins}}_{\mathrm{Bob}}$ and proceeds with the simulated execution of the protocol π by simulating the noiseless messages as well as the inputs/outputs of $F_{P_{V,W|X,Y}}$, and reveals the noiseless messages to A. If the simulated Bob would output $b_{c^{\prime }}^{\prime }$ in the hybrid interaction, then S allows $F_{\mathrm{ROT}}$ to output the bit $b_{c}$.

**Both parties are corrupted:**S just simulates $F_{P_{V,W|X,Y}}$.

We analyze below the probabilities of the events that can result in different views for the environment Z between the real world execution with the protocol π and the adversary A, and the ideal world execution with functionality $F_{\mathrm{ROT}}$ and the simulator S:

- When only Alice is corrupted, Z’s view in the real and ideal worlds are equal if: (1) S succeeds to extract both of Alice’s outputs bits $b_{0},b_{1}$ to forward to $F_{\mathrm{ROT}}$; and (2) A does not learn the choice bit $c^{\prime }$ in the simulated protocol execution. By Lemma 1, the extraction works with overwhelming probability. By the security definitions of Section 3, with overwhelming probability A does not learn $c^{\prime }$.
- When only Bob is corrupted, Z’s view in the real and ideal worlds are equal if: (1) S succeeds to extract the bit *c* and finish the protocol in such way that the received bit $b_{c}^{\prime }$ in the simulated protocol execution is equal to $b_{c}$; and (2) A cannot learn $b_{\bar{c}}^{\prime }$ in the simulated protocol execution. By Lemma 2, the first condition is satisfied with overwhelming probability. By the security definitions of Section 3, with overwhelming probability A cannot learn $b_{\bar{c}}^{\prime }$
- When neither party is corrupted, S’s procedures statistically emulate the hybrid execution for the adversary A, as A cannot learn $b_{0}^{\prime },b_{1}^{\prime },c^{\prime }$ from the noiseless messages alone.
- When both parties are corrupted, S’s procedures perfectly emulate the hybrid execution for the adversary A.

We conclude that, since all events that can result in different views have negligible probabilities, the protocol π UC-realizes $F_{\mathrm{ROT}}$. □

## 5. Conclusions

In this paper, we prove that random oblivious transfer protocols based on two-party stateless functionalities matching a list of security properties are universally composable when unbounded simulators are allowed. As previously commented, this assumption on the simulator gives us secure universal composability with other statistically secure protocols. The restriction to random oblivious transfer protocols is not restrictive (since random OT can be used to obtain OT for arbitrary inputs [11], proving the composability of such reduction is straightforward [12]). Most of the OT protocols based on two-party stateless functionalities are in fact designed to initially run an internal random OT protocol and then derandomize the values. In this case, the universally composability implication can be applied directly to the inner random OT protocol. However, it is an interesting problem to generalize the results presented here to arbitrary OT. Our result immediately implies that several previously proposed OT protocols can have their security upgraded for free [11,13,14,15,16,17,18,19,20,21,22,23,24,25,26].

Finally, we note that, in the case that the extraction (Lemma 1) and equivocability (Lemma 2) procedures can be executed in polynomial time (such as in [11,24]), we achieve fully-fledged UC-security—obtaining secure composability even with protocols that are only computationally secure.
